# Development and Internal Validation of a Novel Nomogram Predicting the Outcome of Salvage Radiation Therapy for Biochemical Recurrence after Radical Prostatectomy in Patients without Metastases on Restaging Prostate-specific Membrane Antigen Positron Emission Tomography/Computed Tomography

**DOI:** 10.1016/j.euros.2024.01.009

**Published:** 2024-02-06

**Authors:** Dennie Meijer, Pim J. van Leeuwen, Wietse S.C. Eppinga, Ben G.L. Vanneste, Philip Meijnen, Laurien A. Daniels, Roderick C.N. van den Bergh, Anne P. Lont, Yves J.L. Bodar, Rosemarijn H. Ettema, Katelijne C.C. de Bie, Frederik H.K. Oudshoorn, Jakko A. Nieuwenhuijzen, Henk G. van der Poel, Maarten L. Donswijk, Martijn W. Heymans, Daniela E. Oprea-Lager, Eva E. Schaake, André N. Vis

**Affiliations:** a Department of Urology, Amsterdam University Medical Center, Prostate Cancer Network Netherlands, Amsterdam, The Netherlands; b Department of Radiology & Nuclear Medicine, Amsterdam University Medical Center, Cancer Center Amsterdam, Amsterdam, The Netherlands; c Department of Urology, The Netherlands Cancer Institute, Prostate Cancer Network Netherlands, Amsterdam, The Netherlands; d Department of Radiation Oncology, University Medical Center Utrecht, Utrecht, The Netherlands; e Department of Radiation Oncology, Maastricht University Medical Center, Maastricht, The Netherlands; f Departments of Radiation Oncology and Human Structure and Repair, Ghent University Hospital, Ghent, Belgium; g Department of Radiation Oncology, Amsterdam University Medical Center, Amsterdam, The Netherlands; h Department of Urology, St. Antonius Hospital, Nieuwegein/Utrecht, The Netherlands; i Department of Urology, Meander Medical Center, Amersfoort, The Netherlands; j Department of Urology, Spaarne Gasthuis, Haarlem, The Netherlands; k Department of Nuclear Medicine, The Netherlands Cancer Institute, Amsterdam, The Netherlands; l Department of Epidemiology and Data Science, Amsterdam University Medical Center, VU University, Amsterdam, The Netherlands; m Department of Radiation Oncology, The Netherlands Cancer Institute, Amsterdam, The Netherlands

**Keywords:** Salvage radiation therapy, Prostate cancer, Prostate-specific membrane antigen imaging, Positron emission tomography/computed tomography, Nomograms

## Abstract

**Background and objective:**

Owing to the greater use of prostate-specific membrane antigen (PSMA) positron emission tomography/computed tomography (PET/CT) in patients with biochemical recurrence (BCR) of prostate cancer (PCa) after robot-assisted radical prostatectomy (RARP), patient selection for local salvage radiation therapy (sRT) has changed. Our objective was to determine the short-term efficacy of sRT in patients with BCR after RARP, and to develop a novel nomogram predicting BCR-free survival after sRT in a nationwide contemporary cohort of patients who underwent PSMA PET/CT before sRT for BCR of PCa, without evidence of metastatic disease.

**Methods:**

All 302 eligible patients undergoing PCa sRT in four reference centers between September 2015 and August 2020 were included. We conducted multivariable logistic regression analysis using a backward elimination procedure to develop a nomogram for predicting biochemical progression of PCa, defined as prostate-specific antigen (PSA) ≥0.2 ng/ml above the post-sRT nadir within 1 yr after sRT.

**Key findings and limitations:**

Biochemical progression of disease within 1 yr after sRT was observed for 56/302 (19%) of the study patients. The final predictive model included PSA at sRT initiation, pathological grade group, surgical margin status, PSA doubling time, presence of local recurrence on PSMA PET/CT, and the presence of biochemical persistence (first PSA result ≥0.1 ng/ml) after RARP. The area under the receiver operating characteristic curve for this model was 0.72 (95% confidence interval 0.64–0.79). Using our nomogram, patients with a predicted risk of >20% had a 30.8% chance of developing biochemical progression within 1 yr after sRT.

**Conclusions:**

Our novel nomogram may facilitate better patient counseling regarding early oncological outcome after sRT. Patients with high risk of biochemical progression may be candidates for more extensive treatment.

**Patient summary:**

We developed a new tool for predicting cancer control outcomes of radiotherapy for patients with recurrence of prostate cancer after surgical removal of their prostate. This tool may help in better counseling of these patients with recurrent cancer regarding their early expected outcome after radiotherapy.

## Take Home Message

We developed a novel nomogram for predicting outcomes of salvage radiation therapy (sRT) after prostate-specific membrane antigen positron emission tomography/computed tomography for patients with biochemical recurrence of prostate cancer after radical prostatectomy. This nomogram may facilitate better counseling of patients regarding early oncological outcomes after sRT. Patients with high risk of developing biochemical progression may be candidates for more extensive treatment.

## 1 Introduction

Radical prostatectomy (RP) is one of the main curative treatment options for patients with localized prostate cancer (PCa). Despite good long-term oncological outcomes, approximately 20–40% of patients experience biochemical recurrence (BCR) after RP [1], [2], [3]. Results from the RAVES, RADICALS, and GETUG AFU-17 randomized clinical trials indicated that (observation followed by) early salvage radiation therapy (sRT) is oncologically noninferior to (immediate) adjuvant RT, but has lower toxicity rates and should therefore be considered a standard of care [4], [5], [6]. As a result, the majority of contemporary patients undergo prostate-specific antigen (PSA) surveillance after RP.

Prostate-specific membrane antigen (PSMA) positron emission tomography/computed tomography (PET/CT) imaging is advised for patients with PSA >0.2 ng/ml and rising after RP and if the results will influence subsequent treatment decisions [7]. Negative PSMA PET/CT findings should not delay sRT if otherwise indicated. Currently, owing to the enhanced detection of metastases with PSMA PET/CT [8], [9], the number of patients with (oligo)-metastatic disease has dramatically increased. In addition, local recurrence of disease is visualized more frequently, guiding potential local salvage treatment strategies such as sRT. However, a substantial number of patients with early BCR of PCa after RP undergo restaging PSMA PET/CT that shows no evidence of disease [10], [11], [12]. In these patients, sRT is also recommended, assuming that they have local recurrent disease. Nomograms that include several biochemical and pathological parameters have been developed to predict sRT outcomes for patients with PCa BCR [13], [14]. However, these nomograms were based on outcomes for patients who did not undergo imaging with modern techniques for restaging, such as PSMA PET/CT. Our group recently demonstrated that a cohort of patients who underwent PSMA PET/CT for restaging before sRT had better short-term oncological outcomes after sRT in comparison to a cohort of patients without PSMA PET/CT before sRT [15]. Consequently, nomograms predicting sRT outcomes that were developed before the introduction of PSMA PET/CT imaging may no longer be as accurate. Our aim was to develop a novel nomogram to predict short-term oncological outcomes for patients who underwent PSMA PET/CT imaging that revealed no metastases before sRT for PCa BCR.

## 2 Patients and methods

### 2.1 Study design and inclusion and exclusion criteria

We evaluated all patients who underwent PSMA PET/CT imaging before sRT to the prostatic fossa for PCa BCR (PSA ≥0.2 ng/ml) in four reference centers for PCa RT in The Netherlands (Amsterdam UMC and Netherlands Cancer Institute, Amsterdam, The Netherlands; University Medical Center Utrecht and MAASTRO Clinic, Maastricht, The Netherlands) between September 2015 and August 2020.

The study was approved by the institutional review board in each participating center (VUmc2019.275; IRBd19182; UMCU21-049; MAASTRO-W-21-02-00060). Patients were not included if they had lymph-node metastases at the time of extended lymph-node dissection during robot-assisted RP (RARP), if there was evidence of metastatic disease on restaging PSMA PET/CT, if they received androgen deprivation therapy or antiandrogen therapy during or before sRT, or if they had insufficient biochemical follow-up (<1 yr after sRT). Patients with local recurrence in the prostatic fossa were eligible for inclusion. For all patients with at least two PSA measurements after RARP, the PSA doubling time was calculated using the Memorial Sloan Kettering Cancer Center online calculator [16].

### 2.2 PSMA PET/CT imaging

All PSMA PET/CT scans were either performed or clinically reviewed in high-volume PCa RT centers according to local protocol. The indication for PSMA PET/CT was PSA ≥0.2 ng/ml. ^18^F-DCFPyL and ^18^F-PSMA-1007 were synthesized via direct radiofluorination at an on-site cyclotron facility, whereas ^68^Ga-PSMA-11 was produced on site in compliance with Good Manufacturing Practice guidelines [17], [18]. PET scans were performed from mid-thigh to the base of the skull, approximately 120 min after injection of a median dose of 293 MBq (interquartile range [IQR] 202–320) for ^18^F-DCFPyL, approximately 60 min after injection of a median dose of 120 MBq (IQR 101–148) for ^68^Ga-PSMA-11, and approximately 90 min after injection of a median dose of 250 MBq (IQR 211–286) for ^18^F-PSMA-1007. PET images were combined with either a low-dose CT scan (120–140 kV, 40–80 mAs with dose modulation) or a diagnostic CT scan (130 kV, 110 mAs) for anatomic correlation and attenuation correction. All PET images were corrected for scatter, decay, and random coincidences.

All PSMA PET/CT scans were discussed in a multidisciplinary meeting attended by at least one highly experienced nuclear medicine physician. In line with the PROMISE criteria, PSMA PET/CT images were assessed for evidence of the presence of local recurrent disease (miTr) [19].

### 2.3 sRT

All patients who underwent sRT received a dose of 60–77 Gy in 20–35 fractions of image-guided RT or volumetric0modulated arc therapy RT to the prostatic fossa. In some cases, if local recurrence was suspected on PSMA PET/CT, a simultaneous integrated boost to the PET-positive lesion was administered. The prostate bed, clinical target volume, and planning target volume were contoured according to the European Organization for Research and Treatment of Cancer guidelines [20]. For all patients, the equivalent dose in 2-Gy fractions (EQD2) was calculated.

### 2.4 Outcome variables and statistical analysis

The study aim was to develop a novel nomogram to predict biochemical progression of disease within 1 yr after sRT without concomitant hormonal treatment in patients who underwent PSMA PET/CT before sRT. Biochemical progression of disease was defined as PSA ≥0.2 ng/ml above the post-RT nadir, or the start of additional treatment after sRT (at the discretion of the treating physician).

Multivariable logistic regression analysis was performed using a backward elimination procedure with a significance level of *p* = 0.157 [21]. PSA at sRT initiation (continuous), pathological grade group (GG; categorical: 1–2 vs 3 vs 4–5), pathological N stage (categorical: pNx vs pN0), pathological T stage (categorical: pT2 vs pT3a vs ≥pT3b), surgical margin status (categorical: negative vs positive), time between RARP and sRT (continuous), PSA doubling time (continuous), PSMA PET/CT findings (categorical: negative vs local recurrence of disease), and biochemical persistence (BCP) after RARP (categorical: no vs yes) were included as potential predictors. The discriminative ability of the model was quantified using the area under the receiver operating characteristic curve (AUC). A calibration plot was generated to assess the agreement between predicted risk and observed risk of biochemical progression within 1 yr after sRT. To this end, patients were grouped by deciles for predicted risk. The average observed risk was then plotted against the average predicted risk for each of the ten groups. Decision curve analysis (DCA) was conducted to visualize the net benefit as a function of the threshold probability. The threshold probability reflects the variation in predicted risk that patients or doctors consider the minimum requirement for undergoing a specific intervention. A higher net benefit for the nomogram means that prediction of biochemical progression within 1 yr after sRT was correct for more patients.

To predict the risk for patients with missing variables, a multiple imputation procedure was used. All model and performance analyses were conducted across the imputed data sets and Rubin’s rules were applied to obtain pooled results [22]. Predicted risk values were subsequently calculated for the complete data sets. Internal validation was performed via bootstrapping with 250 samples to correct for overfitting of the model. For all performance measures, the following steps were applied. First, Rubin’s rules were applied to obtain the pooled coefficients. Second, internal validation was performed to determine the shrinkage factor, which was subsequently applied to the pooled coefficients. These coefficients were then used to calculate the performance metrics (eg, AUC, DCA). Analyses were performed in RStudio v4.1.2 (R Foundation for Statistical Computing, Vienna, Austria) with the *mice* and *psfmi* packages [22].

## 3 Results

### 3.1 Baseline characteristics

We included 302 patients who underwent PSMA PET/CT before sRT at a median age of 68 yr (IQR 64–72). The median time between RARP and restaging PSMA PET/CT was 26 mo (IQR 14–56; Table 1). According to the European Association of Urology (EAU) BCR risk categories, 62/302 patients (21%) were considered low risk and 183 (60%) were considered high risk; the risk category could not be calculated for 57 patients (19%) because of missing data for pathological GG or PSA doubling time.

**Table 1:** Baseline characteristics of 302 patients treated with sRT after PSMA PET/CT imaging

Parameter	Result
Median age at sRT, yr (IQR)	68 (64–72)
Median PSA at sRT initiation, ng/ml (IQR)	0.3 (0.2–0.5)
Median total dose administered, Gy (IQR)	70 (66–70)
Median EQD2, Gy (IQR)	70 (70–70)
Median interval between RARP and sRT, mo (IQR)	26 (14–56)
Median PSA doubling time, mo (IQR)	7.8 (4.0–14.7)
EAU BCR risk category, *n* (%)	
Low risk	62 (21)
High risk	183 (60)
Data missing	57 (19)
Pathological ISUP grade group, *n* (%)	
Grade group 1	32 (11)
Grade group 2	117 (39)
Grade group 3	77 (25)
Grade group 4	36 (12)
Grade group 5	36 (12)
Data missing	4 (1)
Pathological T stage, *n* (%)	
≤pT2	152 (51)
pT3a	103 (34)
≥pT3b	46 (15)
Data missing	1 (<1)
Surgical margin status, *n* (%)	
Negative	140 (46)
Positive	157 (52)
Data missing	5 (2)
Biochemical persistence after RARP, *n* (%)	
No	226 (75)
Yes	73 (24)
Data missing	3 (1)
Restaging PSMA PET/CT findings, *n* (%)	
Negative for cancer	241 (80)
Local recurrence of disease	61 (20)

sRT = salvage radiation therapy; IQR = interquartile range; PSA = prostate-specific antigen; RARP = robot-assisted radical prostatectomy; EAU = European Association of Urology; BCR = biochemical recurrence; ISUP = International Society of Urological Pathology; PSMA = prostate-specific membrane antigen; PET = positron emission tomography; CT = computed tomography; EQ2D = equivalent dose in 2-Gy fractions

### 3.2 PSMA PET/CT findings

PSMA PET/CT was negative for cancer in 241/302 patients (80%) but revealed local recurrent disease (miTr) in 61/302 patients (20%; Table 1). In the group with PSA <0.5 ng/ml at PSMA PET/CT, 12% (27/223 patients) had local recurrent disease, compared to 43% (34/79 patients) in the group with PSA ≥0.5 ng/ml at PSMA PET/CT (*p* < 0.001).

### 3.3 sRT

All 302 patients who were included underwent sRT to the prostatic fossa, which was initiated at median PSA of 0.3 ng/ml (IQR 0.2–0.5; Table 1). No concomitant hormonal therapy was administered (exclusion criterion). A total of 70 Gy (IQR 66–70) was administered, fractionated over a median of 35 (IQR 30–35) sessions. Of all the patients included, 56/302 (19%) experienced biochemical progression within 1 yr after sRT. Patients with local recurrent disease on PSMA PET/CT had biochemical progression in 16% of cases (10/61 patients), compared to 46/241 patients (19%) of patients with a negative for cancer PSMA PET/CT (*p* = 0.6). The proportion of patients with biochemical progression within 1 yr after sRT was 5% (5/62 patients) in the EAU low-risk group and 23% (42/183 patients) in the EAU high-risk group (*p* = 0.01). The proportion of patients with biochemical progression 1 yr after sRT was 9% (eight/87 patients) in the group with PSA ≤0.2 ng/ml before sRT, 20% (30/149 patients) in the group with PSA of 0.3–0.5 ng/ml, and 27% (18/66 patients) in the group with PSA ≥0.6 ng/ml (*p* = 0.013).

### 3.4 Novel prediction model

PSA at sRT initiation (continuous), pathological GG (categorical: 1–2 vs 3 vs 4–5), surgical margin status (categorical: negative vs positive), PSA doubling time (continuous), local recurrence on PSMA PET/CT (categorical: negative vs miTr), and BCP after RARP (categorical: no vs yes) were included in the final model (Table 2). The AUC for this model was 0.72 (95% confidence interval [CI] 0.64–0.79; Supplementary Table 1). The novel nomogram is freely available online (https://www.evidencio.com/models/show/3019).

**Table 2:** Multivariable logistic regression analysis for prediction of biochemical progression at 1 yr after sRT in 302 patients who underwent PSMA PET/CT before sRT

Variable	OR (95% CI)	*p* value
Pathological ISUP grade group		
Grade group 1–2	Reference	
Grade group 3	2.02 (0.92–4.40)	0.078
Grade group 4–5	2.88 (1.35–6.15)	0.006
Positive surgical margin status (vs negative)	0.53 (0.28–1.03)	0.062
Biochemical persistence after RARP (vs no persistence)	1.82 (0.91–3.64)	0.088
log_2_ (PSA doubling time)	0.73 (0.55–0.97)	0.029
log_2_ (PSA at sRT initiation)	1.41 (1.03–1.93)	0.031
Restaging PSMA PET/CT positive for local recurrence of disease (vs negative)	0.52 (0.21–1.29)	0.156
Model AUC (95%CI)	0.72 (0.64–0.79)	

OR = odds ratio; CI = confidence interval; ISUP = International Society of Urological Pathology; RARP = robot-assisted radical prostatectomy; PSA = prostate-specific antigen; PSMA = prostate-specific membrane antigen; PET = positron emission tomography; CT = computed tomography; sRT = salvage radiation therapy; AUC = area under the receiver operating characteristic curve

**Fig. 1:**
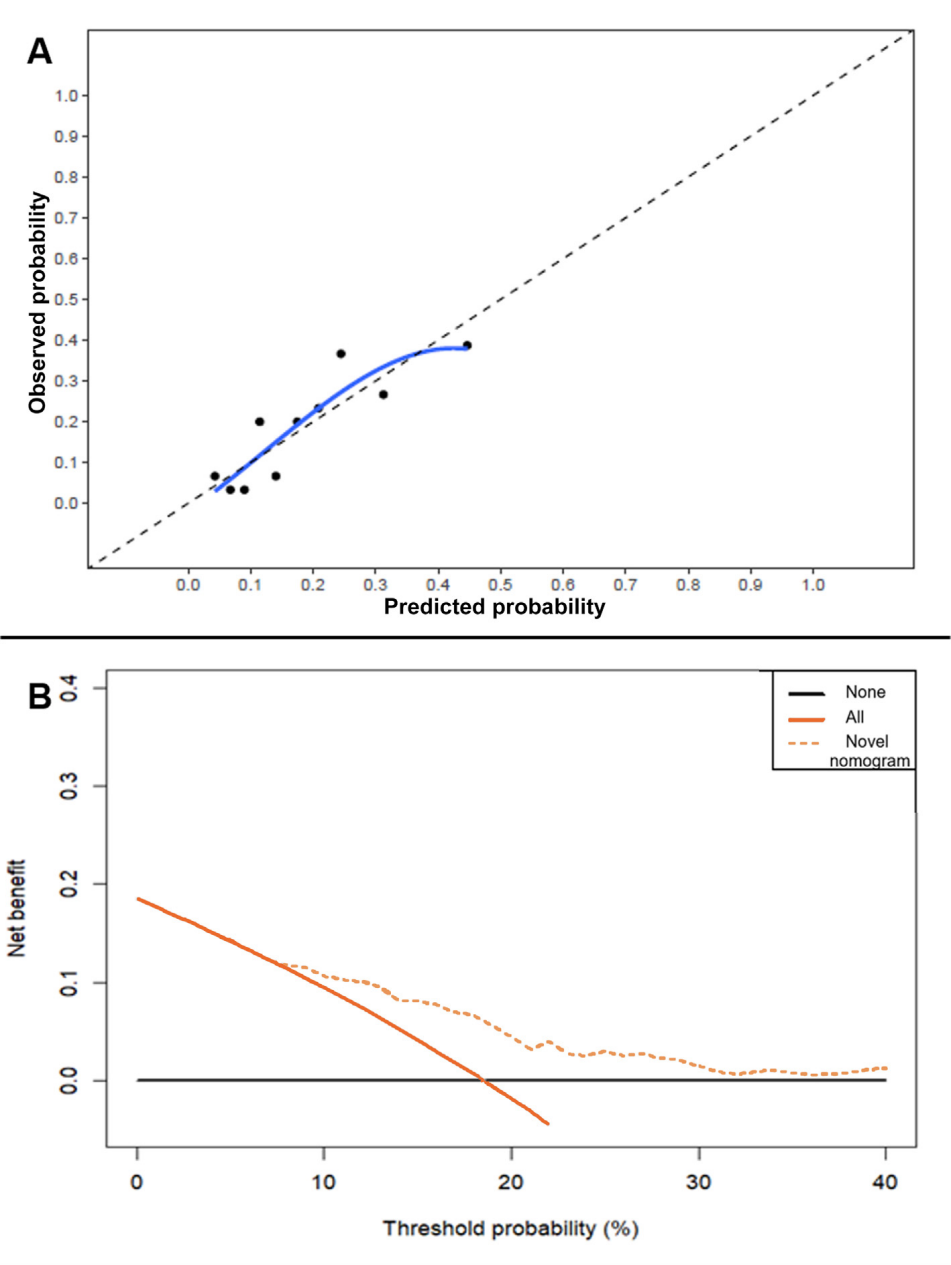
(A) Calibration plot and (B) decision curve analysis for the novel nomogram.

The calibration plot in Figure 1A demonstrates that the nomogram is well calibrated, with good correlation between predicted and observed outcome. The range for the predicted values is 0–50%, with explained variance (R^2^) of 0.151. DCA revealed that the nomogram yielded an increase in net benefit at a threshold probability of ≥4% (Fig. 1B).

### 3.5 Clinical applicability

Among patients with a predicted risk of <10% of developing biochemical progression after sRT, only three of 70 (4.3%) actually experienced biochemical progression 1 yr after sRT (Table 3). Conversely, among patients with a predicted risk of >20% according to the novel nomogram, 36/117 (30.8%) actually experienced biochemical progression 1 yr after sRT.

**Table 3:** Proportion of patients experiencing biochemical progression according to different hypothetical cutoffs for the novel nomogram on when to perform salvage radiation therapy to the prostatic fossa without concomitant hormonal therapy

Predicted risk		Patients with biochemical	
Threshold, %	Patients,	progression, *n* (%)	
	*n* (%)	No	Yes
0–10	70 (23)	67 (96)	3 (4.3)
>10	232 (77)	179 (77)	53 (22.8)
>20	117 (39)	81 (69)	36 (30.8)
>30	44 (15)	28 (64)	16 (36.4)
>40	11 (4)	4 (36)	7 (63.6)

### 3.6 Internal validation

The regression coefficients for the final model were multiplied by a shrinkage factor of 0.817, as derived from bootstrapping. The optimism-corrected AUC and R^2^ variance for the model are 0.681 and 0.095, respectively.

## 4 Discussion

Traditional imaging modalities are unreliable in excluding concurrent systemic disease in patients with rising PSA. Given the side effects of sRT and the reported reduction in health-related quality of life [23], [24], [25], there is reluctance to offer local salvage treatment that may ultimately prove futile in the presence of coexisting metastatic disease. Besides, in patients with measurable but nonprogressing PSA after RP, sRT can be considered as overtreatment. Nevertheless, sRT is recommended even in the absence of abnormalities on imaging. As PSMA PET/CT has greater diagnostic accuracy for detection of metastatic disease in comparison to conventional imaging techniques (92% vs 65% in the proPSMA-study [8]), it may have a substantial influence on management decisions for patients with BCR [26], [27].

We developed a novel nomogram for prediction of biochemical progression of disease within 1 yr after sRT in patients with BCR PCa who underwent PSMA PET/CT imaging for restaging. The final predictive model included PSA at sRT initiation, pathological GG, surgical margin status, PSA doubling time, PSMA PET/CT findings, and BCP after RARP. The AUC for this model was 0.72 (95% CI 0.64–0.79).

As PSMA PET/CT imaging has only been routinely performed in patients with BCR after RARP for a few years, our selection of the outcome variable for the predictive nomogram was limited to biochemical progression within 1 yr after sRT. In the ongoing PSMA-SRT randomized trial [28], PSMA PET/CT was able to detect disease outside the prostatic fossa in 25% (26/103) of patients. Historically, without PSMA PET/CT imaging for restaging, these patients would probably experience biochemical progression after local salvage treatment. In the present study, we only included patients without metastatic disease on PSMA PET/CT. Hence, we believe that our biochemical progression rate within 1 yr after sRT (19%) might not increase substantially after a longer follow-up period and is therefore an accurate surrogate for long-term biochemical progression.

All current nomograms for predicting oncological outcomes after sRT were developed before the introduction of PSMA PET/CT. Stephenson et al [14] developed a nomogram predicting the probability of 6-yr biochemical progression–free survival after sRT in a multi-institutional cohort of 1540 patients. Their nomogram, which includes 11 parameters such as surgical margin status and PSA doubling time, had an AUC of 0.69. Campbell et al [13] developed a model that includes 12 parameters in a cohort of 1005 patients and obtained an AUC of 0.74 for predicting BCR at 5 yr after sRT. In the present study, the AUC for prediction of biochemical progression after sRT was 0.72. As we used a standardized method for selecting variables, pathological T stage and the time between RARP and sRT, for instance, were not included in the final model. Although these potential prognostic variables were not associated with our outcome variable and were therefore not included in the final model consisting of six parameters, it can be hypothesized that they would slightly increase the predictive ability of the nomogram. However, we chose to increase the clinical applicability of the model and therefore excluded variables that were not (significantly) associated with the outcome variable.

Zamboglou et al [29] recently evaluated metastasis-free survival and patterns of metastatic disease in patients undergoing PSMA PET/CT–guided sRT. They found that patients with PET-positive lymph nodes on PSMA PET/CT had significantly worse oncological outcomes than patients without metastatic disease on PSMA PET/CT. In the present study, we excluded patients with metastatic disease on PSMA PET/CT from the analysis. Patients with metastatic disease on restaging PSMA PET/CT are probably more likely to experience recurrence after local salvage treatment. To date, however, it is not known whether local sRT in patients with metastatic disease on PSMA PET/CT improves oncological outcomes in comparison to no local salvage treatment. Further prospective trials are warranted to investigate the role of local salvage treatment in patients with (oligo-)metastatic disease.

We feel that use of a predictive nomogram can add to counseling for patients with BCR after RARP. For the group of patients predicted to have <10% risk of developing biochemical progression according to our novel nomogram, only 4.3% had experienced biochemical progression 1 yr after sRT. On the basis of these findings, administration of sRT without concomitant hormonal therapy should be encouraged. As we did not include patients who did not receive any sRT, we were unable to compare these sRT outcomes with the natural history of PSA progression in patients undergoing surveillance instead of immediate sRT after RARP. Among patients predicted to have >20% risk of developing biochemical progression according to our nomogram, 30.8% actually experienced biochemical progression within 1 yr after sRT. It may be worth investigating whether a more extensive radiation treatment strategy or addition of concomitant hormonal treatment, as proposed by Pollack et al [30] and Spratt et al [31], would reduce the risk of disease progression in these patients.

Our study is not free of limitations. First, the retrospective nature may have potentially introduced patient selection biases. Second, it should be noted that PSMA PET/CT scans were reported as part of routine clinical practice and were not part of a prospective clinical trial or centrally reviewed. Therefore, different scan protocols, PSMA PET tracers, and PET scanners were used. Third, there were minor differences in the total sRT dose and the number of fractions between the participating centers. Since the target volume was defined according to the EORTC guidelines [20] and the EQD2 is similar, the study is not hampered by the inclusion of patients with extended radiation fields. Fourth, we included a relatively small number of patients and did not externally validate our novel nomogram, which will be of great importance before applying the nomogram in clinical practice. Finally, as BCR of PCa after sRT is not directly correlated with metastases-free or overall survival, it may not be a reliable surrogate for long-term oncological outcomes.

## 5 Conclusions

We developed a novel nomogram for prediction of biochemical progression of disease within 1 yr after sRT in patients with BCR of PCa who underwent PSMA PET/CT imaging for restaging purposes, without evidence of metastatic disease. To the best of our knowledge, this is the first nomogram to include PSMA PET/CT findings for patients undergoing sRT. The novel nomogram shows good performance and could result in better counseling of patients regarding oncological outcomes after sRT and thus more informed decision-making.

  ***Author contributions***: Dennie Meijer had full access to all the data in the study and takes responsibility for the integrity of the data and the accuracy of the data analysis.

  *Study concept and design*: Meijer, van Leeuwen, Vis.

*Acquisition of data*: Meijer, van Leeuwen, Eppinga, Vanneste, Meijnen, Daniels, van den Bergh, Lont, Bodar, Ettema, de Bie, Oudshoorn, Schaake, Vis.

*Analysis and interpretation of data*: Meijer, van Leeuwen, Eppinga, van den Bergh, Vis.

*Drafting of the manuscript*: Meijer, van Leeuwen, Eppinga, van den Bergh, Vis.

*Critical revision of the manuscript for important intellectual content*: Vanneste, Meijnen, Daniels, van den Bergh, Lont, Bodar, Ettema, de Bie, Oudshoorn, Nieuwenhuijzen, van der Poel, Donswijk, Heymans, Oprea-Lager, Schaake.

*Statistical analysis*: Meijer, van Leeuwen, van den Bergh, Heymans, Vis.

*Obtaining funding*: None.

*Administrative, technical, or material support*: None.

*Supervision*: van Leeuwen, Eppinga, van den Bergh, Vis.

*Other*: None.

  ***Financial disclosures***: Dennie Meijer certifies that all conflicts of interest, including specific financial interests and relationships and affiliations relevant to the subject matter or materials discussed in the manuscript (eg, employment/affiliation, grants or funding, consultancies, honoraria, stock ownership or options, expert testimony, royalties, or patents filed, received, or pending), are the following: None.

  ***Funding/Support and role of the sponsor***: None.
