# Supplementary material for: Development and Internal Validation of a Novel Nomogram Predicting the Outcome of Salvage Radiation Therapy for Biochemical Recurrence after Radical Prostatectomy in Patients without Metastases on Restaging Prostate-specific Membrane Antigen Positron Emission Tomography/Computed Tomography
Source: Eur Urol Open Sci. 2024 Feb 6;61:37–43. doi: 10.1016/j.euros.2024.01.009 (PMC10879939; doi:10.1016/j.euros.2024.01.009)
Supplement: Supplementary data 1 [file mmc1.docx]

**Supplementary Table 1.** Coefficients of the novel nomogram

| **Variable** | **Beta coefficient** |
| --- | --- |
| Intercept | -0.449199 |
| Pre-SRT PSA-value | 0.3453365 |
| PSA doubling time | -0.3116610 |
| Pathological GG 3 | 0.7016797 |
| Pathological GG 4-5 | 1.057292 |
| Positive surgical margin | -0.6270813 |
| BCP after RARP | 0.6003587 |
| Local recurrence on PSMA | -0.6545496 |
